# Laparoscopic intraperitoneal onlay mesh for pediatric incisional hernia—a case report

**DOI:** 10.1186/s40792-017-0400-5

**Published:** 2017-12-07

**Authors:** Maho Inoue, Shigeyoshi Aoi, Akihiro Taniguchi, Kohei Sakai, Mayumi Higashi, Shigehisa Fumino, Taizo Furukawa, Tatsuro Tajiri

**Affiliations:** Department of Pediatric Surgery, Kyoto Prefectural University of Medicine, 465 Hirokoji-Kamigyoku, Kyoto, 602-8566 Japan

**Keywords:** Incisional hernia, Laparoscopic surgery, Intra peritoneal onlay mesh, Pediatric surgery

## Abstract

**Background:**

The incidence of incisional hernia in pediatric patients is low in comparison with that reported in adults. In the pediatric population, primary closure has generally been favored. However, synthetic or biomedical mesh offers advantages in the repair of larger defects when primary closure is difficult. The use of laparoscopic intraperitoneal onlay mesh (IPOM) in the adult population has been well documented. In the pediatric population, a few laparoscopic approaches with direct suturing have been proposed; however, there are no reports of laparoscopic repair with the use of IPOM.

**Case presentation:**

The patient was a 1-year-old girl with epigastric incisional hernia after an operation to correct a complete arteriovenous septal defect. The fascial defect (size 30 × 35 mm) was large; thus, direct suturing was considered to be associated with a high risk of thoracic deformation and recurrence.

Laparoscopic IPOM was performed. The fascial defect was detected precisely through the laparoscopy, and non-absorbable mesh was placed through a 12-mm trocar. Minimal incisions were required for the trocars, and extensive dissection of the abdominal wall structure was not needed. This procedure allowed for the integrity and functional status of the abdominal wall to be maintained.

**Conclusion:**

Laparoscopic IPOM is a minimally invasive and cosmetically acceptable method that can be applied to the treatment of large incisional hernias in children.

## Background

The incidence of incisional hernia among children undergoing primary abdominal surgery at < 6 months of age is reported to be 2.3% [1]. This incidence is low in comparison with that reported in adults (10–50%) [2]. In the pediatric population, primary closure has generally been favored. A few laparoscopic approaches with direct suturing have been proposed [3, 4]; however, there have been no reports of laparoscopic repair with the use of mesh in the pediatric population. Direct repair without mesh is associated with high rates of recurrence in adults (direct suture, 12–54%; tension-free repair, 2–36%) [5]. Thus, the use of mesh reinforcement has been encouraged to relieve tension on the fascial repair [6, 7]. The application of laparoscopic intraperitoneal onlay mesh (IPOM) procedures in adult patients has been well documented. In the present study, we describe the use of laparoscopic IPOM in the treatment of a 1-year-old girl with a large epigastric incisional hernia after corrective surgery to treat a complete arteriovenous septal defect.

## Case presentation

**Fig. 1:**
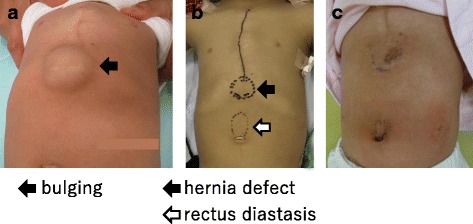
The physical examinations before (**a**, **b**) and after (**c**) the operation. The hernia defect measured 30 × 35 mm, located on 5 mm caudal to the xiphoid process. The rectal diastasis, which was detected 5 mm cranial to the umbilicus, is marked (**b**)

**Fig. 2:**
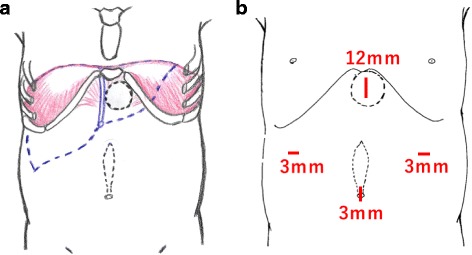
The location of the defect and port setting. The defect was adjacent to the diaphragm; the liver was located just under the defect (**a**). In total, four ports were placed in the abdomen (**b**)

**Fig. 3:**
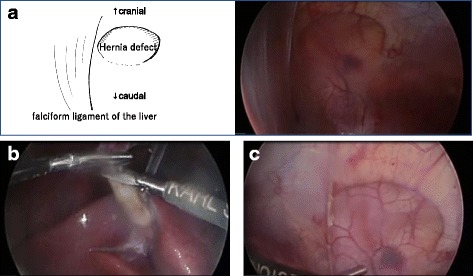
Exposure of the hernia defect. The fascial defect was adjacent to the falciform ligamentum of the liver (**a**). The falciform ligamentum was incised with scissors after coagulation (**b**, **c**)

**Fig. 4:**
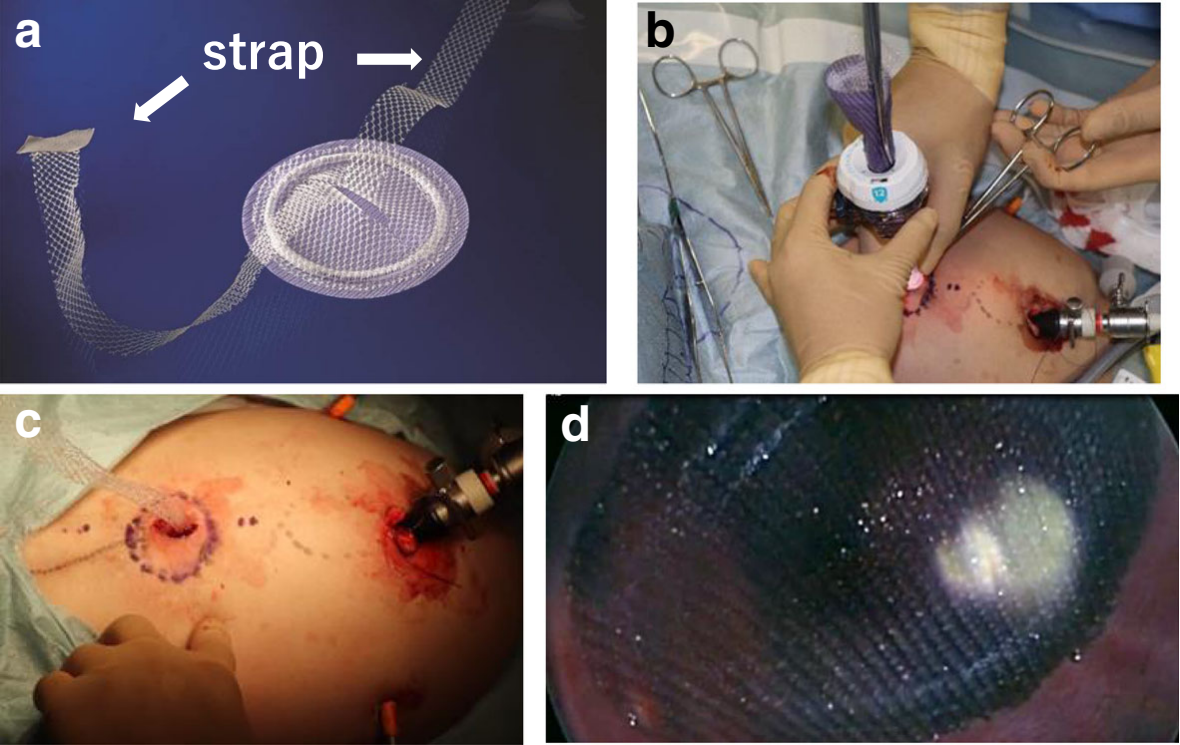
Mesh insertion and placement. A rolled-up 8.0 × 8.0 cm Bard®Ventralrex®ST (L) was inserted through a 12-mm ENDOPATH®XCEL BLADLESS trocar (**a**, **b**). While the strap pulling the strap against the abdominal wall after extracting the trocar (**c**), the mesh was unrolled and placed on the hernia defect intraperitoneally (**d**)

The patient was a 1-year-old girl with trisomy 21 who was diagnosed with complete arteriovenous septal defect (cAVSD) at birth and who had undergone pulmonary artery banding and ligation of the patent ductus arteriosus at 42 days of age. cAVSD repair and pulmonary artery debanding and plasty were performed 1 year later. A bulge in the epigastric abdominal wall was noticed after these operations. The patient had no history of incarceration. An abdominal protrusion measuring 30 × 35 mm in size was located 5 mm caudal to the xiphoid process when the patient was in a supine position (Fig. 1). An intra-umbilical incision was made, and a 3-mm trocar was inserted. Pneumoperitoneum (8 mmHg) was maintained using carbon dioxide insufflation. A 3-mm 30° laparoscope was introduced, and the abdominal wall was inspected. Three-millimeter trocars were inserted in the bilateral flank, as shown in Fig. 2b. The fascial defect was exposed by the left side of the falciform ligament of the liver as shown in Fig. 3a. The falciform ligament was incised to create an adequate space for the placement of the mesh, as shown in Fig. 3c. The size of the mesh was decided based on the size of the hernia orifice, with 2.5 cm of extra coverage on each side. We applied an 8.0 × 8.0 cm Bard®Ventralrex®ST (L), which was coated with hyaluronic acid sodium and carboxy methyl cellulose. A 12-mm ENDOPATH®XCEL BLADLESS trocar was inserted into the hernia orifice, and rolled-up mesh was inserted through the trocar as shown in Fig. 4. After extracting the trocar, the mesh was unrolled and placed on the hernia defect intraperitoneally, while pulling the strap against the abdominal wall. Through the extended first umbilical incision, the rectus diastasis was closed using the open method, and the caudal part of the mesh was sutured directly to the fascia at the same time. Fixation methods (include the use of tackers or percutaneous suture fixation) were not applied to avoid pneumothorax and vascular injuries. The recovery was uneventful. Oral feeding was started on postoperative day 2. The patient was discharged on postoperative day 6. There has been no evidence of recurrence or complications in the 2 months since surgery.

### Discussion

The IPOM technique allows for the placement of a large mesh through a 12-mm-diameter working port to cover a hernia defect with an adequate mesh margin. The intraperitoneal placement of the mesh intraperitoneally avoids extensive tissue dissection. As a result, IPOM reduces the chance of surgical-site infection [8]. This technique, which does not require a major abdominal incision, allows for the integrity and the functional status to be maintained. A hernia defect of > 10 cm in size is a significant risk factor for recurrence according to the adult guidelines [8]. In our case, the risk of recurrence was assumed to be high because the 30 × 35 mm defect in the 8-kg patient was considered to be large relative to an adult of 60 kg in body weight. Moreover, it was likely that direct suturing would have caused thoracic deformation and compression of the heart. Thus, mesh repair was finally performed. A Bard®Ventralrex®ST mesh, which has a strap for pulling up and fixing the mesh to the abdominal wall, was applied. Traction, which was applied using the strap, and the recoiling polydioxanone structure of the mesh provided complete expansion. One of greatest merits of laparoscopic surgery in pediatric cases is that the magnified view helps in deciding the best treatment when the objective is too small to approach directly. In terms of complications, cases of iatrogenic enterotomy, which is associated with the need for extensive adhesiolysis, have been reported [9]. Many studies have noted that mesh-induced visceral complications, which include adhesion, fistulation, and migration of the mesh, should be considered to be very important postoperative concerns [10]. Although various types of meshes have been developed, none has been able to completely suppress tissue reactions [11]. In this case, the risk of adhesion to the intestine was not considered highly, because the liver was located just below the mesh. The preperitoneal onlay mesh (PPOM) repair, dissecting extraperitoneally, has been reported in adult cases in these days [12]. Although, to eliminate the visceral organ’s adverse chronic reactions to the synthetic mesh, placing synthetic mesh in the preperitoneal place may be ideal, the creation of the preperitoneal pocket for mesh placement might be technically complicated and time-consuming in the pediatric small abdominal cavity like this case. It is difficult to know the exact biological processes that occur as a result of the long-term placement of the mesh in the peritoneal cavity of infants. For example, multiple studies have shown that patients who undergo congenital diaphragmatic hernia repair with a prosthetic patch have an increased incidence of chest wall deformities [13–15]. Long-term follow-up is recommended to ascertain how the mesh affects development.

## Conclusions

Laparoscopic IPOM is minimally invasive and cosmetically acceptable method for repairing large incisional hernias in children.

## Abbreviations

cAVSD: Complete arteriovenous septal defect
IPOM: Intraperitoneal onlay mesh
